# A Hierarchy of Deaths: Stem Cells, Animals and Humans Understood by Developmental Biologists

**DOI:** 10.1080/09505431.2019.1579787

**Published:** 2019-03-05

**Authors:** Noémie Merleau-Ponty

**Affiliations:** Reproductive Sociology Research Group (ReproSoc), University of Cambridge, Cambridge, UK

**Keywords:** Stem cells, animal, human, developmental biology, life, death

## Abstract

Stem cell basic science has sparked a lot of attention because of its use of cells coming from ‘destroyed’ embryos. An ethnographic study conducted in two developmental biology laboratories located in India and France demonstrates that lab professionals do not see the use of these cells as controversial. What appears to be a major topic of reflection is the killing of mice. A hierarchy of deaths is delineated when biologists evoke the kind of lives at play in their science. A comparison between narrations of cell experimentations and mice sacrifices enriches a biological approach to the living through genetics, which is nonetheless performed in daily scientific practices. Laboratory workers enact other perceptions that point at being alive or having a life. They acknowledge, with personal convictions or expressions of intense affects, lives that are said to be embodied and experienced, while being hierarchised for the sake of science and dying patients. Laboratory workers’ narratives of a hierarchy of deaths provide them with arguments to engage with discussions happening outside of their workplace about the handling of living materials in experimental settings.

## Introduction

Basic research in developmental biology using human embryonic stem cells (huES) has sparked a lot of attention since the 2000s because these cells can come from human embryos once conceived in reproductive medicine units. Developmental biology studies the growth of tissues after fertilisation and has benefited from the invention of *in vitro* fertilisation, a technique used successfully since 1978 to conceive embryos in laboratories. One outcome of such technique is the production of supernumerary embryos that can be frozen and are sometimes given to basic research. A topic of discussion stemming from such donations is the ‘destruction’ of embryos in the process of making cell lines out of them. Such research also accompany narratives of hopes for new cures through the invention of a new medicine, the regenerative one. Incurable and lethal diseases could be treated if new cell therapies were to be invented. More generally, the use of animals in basic research is also source of intense controversial discussions within and outside of scientific communities. In developmental biology, animals are commonly used to conceive embryos, and gestating mice, for example, are ‘sacrificed’ to study the molecular mechanisms implied in the growth of embryonic and foetal cells, or to study mutations that are implied in various pathologies.

These three examples (destruction of embryos, lethal diseases and animal sacrifices) point at the presence of death in the making of the science of life. How do developmental biologists talk about death in their activities? What does death reveal about the ways biologists understand the lives of the materials they work (cells and mice)?

In this article, I draw upon narratives about the therapeutic horizon of research and the different cells, embryos and mice eleven laboratory practitioners experiment on. Located in France and in India, these biologists express similar narratives based on shared practices, while they acknowledge local variations of societal debates. I share their meanings and emotions associated with the deaths of stem cells, mice and patients. In these narratives, deaths are expressed through various understandings of life. Biology – their science of life – is one major aspect of these understandings but it is not the only one. Biologists intertwine their science with personal convictions or religious beliefs that differentiate being alive and living a life, being an animal and belonging to ‘humanity’. These convictions are strongly connected to the meanings associated with dying and killing.

Building on feminist writings about death in the life sciences, with a focus on animal sacrifices and the care for cells, in this article I also discuss topics such as the molecularisation of the living and the embodiment of the lived. I argue that laboratory workers delineate a hierarchy of deaths that is dependent on how they understand what kinds of lives are handled in the context of basic science in developmental biology.

## Death and the Science of Life

‘Life becomes imaginable as always pregnant with death’ (Bharadwaj and Inhorn, 2016, p. 67). Writing on reproductive medicine and stem cell research, Aditya Bharadwaj and Marcia Inhorn underline various deaths accompanying biotechnologies such as embryo loss in *in vitro* fertilization, embryo donation to basic research or foetal reduction following multiple pregnancies. They draw on a body of literature that has extensively shown how death is central to an anthropology of the biosciences, reproduction and health (Cecil, 1996; Layne, 2002; Lock, 2002; Franklin and Lock, 2003).

In biology laboratories, death is also part of making the knowledge of life. The most studied aspect of death in basic science is animal sacrifices. The study of animal sacrifices reveals ways of understanding animal lives and how they are cared for. The literature on cell culture shows common traits with the ways cells are also used and cared for. Finally, death also appears under the trait of genetic mutation and human lethal diseases.

### Animal Sacrifices

Animal sacrifices are one major expression of death in the lab and has been studied from different perspectives. The literature on animal research has characterised sacrifices as the transformations of animals into generalisable knowledge for science, understood as the common and valuable goal of experimentations (Arluke, 1988; Lynch, 1988; Haraway, 1997; Rémy, 2009; Thompson, 2013). Relating to classical studies, these events enact situations in which a life considered to be of lesser worth is taken to obtain a greater good (Hubert and Mauss, 1899; Durkheim, 2008). In the case of animal research, an animal’s life is taken instead of a human one, as a substitutive subject sharing a common biological corporeality, in the pursuit of new cures for patients (Rémy, 2009; Thompson, 2013, chapter 6). The literature on animal sacrifices shows an ambiguous back and forth logic that identifies and differentiates lab animals and human beings.

Writing on scientific experiments, Birke *et al*. (2007) underline numerous ways of defining animals. Amongst the various significances at play, a dialectic of similarities and differences between animals and humans is stressed as both belong to the ‘animal kingdom’ from a biological perspective but are also different ‘kinds’ (Birke *et al*., 2007, p. 19). The ways humans relate to different animal kinds vary in time and space, through diverse cultures and various forms of encounters. The ‘lab animal’ (Birke *et al*., 2007, p. 21) is one form of the animal kind (with variations depending on the species). ‘A lab animal, after all, is one whose life is determined by the requirements of science’ (Birke *et al*., 2007, p. 25).

Donna Haraway (2008) explored what relating to a lab animal means when she wrote about the ‘politeness’ implied in the ‘instrumental relations’ embedded in care and shared suffering. Finding similarities between lab animals being sacrificed and scapegoats, she writes:

The substitute, the scapegoat, is not Man but Animal. Sacrifice works; there is a whole world of those who can be killed, because finally they are only something, not somebody, close enough to “being” in order to be a model, substitute, sufficiently self-similar and so nourishing food, but not close enough to compel response. (Haraway, 2008, p. 79)

This back and forth logic of closeness and distance has also been analysed by Tora Holmberg through an emphasis on the emotions associated with killing as a form of ‘mortal love’. Mortal love encompasses dilemmas engrafted in the ‘dialectics of care and exploitation’ and the importance of ‘killing well’ (Holmberg, 2011, p. 148 and 154). This ambiguous relationship is also highlighted by Svendsen and Koch (2013) who write about the sacrifices of piglets in experimental neonatal research. They stress that ‘a calculative exchange that defines an absolute moral difference between humanity and animality and constitutes the piglet as a raw material of science, and a corporeal exchange by which the piglet appears as a sentient substitute belonging to the same collective as the researchers’ (Svendsen and Koch, 2013, p. S119). Lesley Sharp (2017) has also written about the links between morality and identification, when describing the ‘humanization’ of monkeys as they are given access to television in order to entertain themselves in their scientific homes.

If sacrifices imply that animals are treated as instruments for the sake of science, requirements of science entail ‘humane’ practices of care that are not only expressed by subjective and affective accounts of biologists but are also historically situated. In the UK, for example, the notion of ‘animal welfare’ has been central to the regulation of animal experimental use (Kirk, 2010, 2014, 2018), and interdisciplinary collaboration explicitly addresses how the science of life is not to be radically differentiated from the humanities in that respect (Davies *et al*., 2016).

### Caring for the Cells

Focusing on the daily activities of stem cell culture, similarities with animal experimentation and their dialectic of care and instrumentation can also be found. In the USA, following a model of organ donation, biologists have tried to develop a framework to assess the ‘death’ of embryos as a moral marker of their donation to basic science and their transformation into stem cell lines (Testa, 2008). Strong emotions and feelings of attachments can also be associated with the daily scientific cultures of stem cells (Suzuki, 2015; Merleau-Ponty, 2018a).

Writing on the ways biologists connect with cells while they culture them in experimental protocols, Mianna Meskus (2018, chapter 5) refers to a ‘continuum of instrumentality and care’ in the ‘crafting’ of a new stem cell biotechnology (induced pluripotent stem cells). Looking at the practical and embodied routine of handling cells in the laboratory she finds that ‘all forms of instrumentalisation are not destructive, and all forms of care are not without the intention of benefit’ (Meskus, 2018, p. 150). One benefit implied in the care for animals and cells is the making of science and its applicative potentials, often referred as ‘translation’ (Friese, 2013).

### Projecting Biomedical Applications

Similarly to animal experimentation, the life and death of cells are related to the life and death of patients who suffer from lethal diseases. Rayna Rapp showed how cells cultured in laboratories and patients waiting for cures are connected through genetic mutations (Rapp, 2003, p. 135). Death, in such cases, is associated with the study of genetic mutations that can be lethal for human patients. Death intertwines basic science and its applicative therapeutic horizons. It is linked to an interpretation of life as being ‘itself’. Writing from her reading of Canguilhem and Foucault, Franklin (2000) analyses that ‘life itself’ is a genetic code, a molecular approach to life, a process of signification inscribed in matter. ‘Nature becomes biology becomes genetics, through which life itself becomes reprogrammable information’ (Franklin, 2000, p. 190). This biological approach to life and medicine relates cells, animals, patients and biologist united in a common goal: increasing scientific knowledge in pursuit of therapeutic applications. This said, when looking at how the death of cells, animals and humans are expressed, this common goal entails a strong hierarchy stemming from different ways of valuing lives.

### A Hierarchy of Deaths for Different Kinds of Lives

As the empirical findings in this article show, whilst producing a knowledge of ‘life itself’, laboratory workers link together patients’ deaths, animal deaths and cell death by pointing at embodied, experienced understandings of life. Responding to a molecular based interpretation of life, Didier Fassin (2009) suggested focusing on lived experiences of being alive. ‘Life which is lived through a body (not only through cells) and as a society (not only as species). I propose to name it “life as such”. Obviously it is related through many ramifications to “life itself” if we use this expression to designate the biological existence of the living and its political extension as populations’ (Fassin, 2009, p. 48). I am not investigating populations here, but nonetheless find common features with my ethnography.

Technobiological understandings of life as being itself are to be found along understandings of life as being embodied in existences that are hierarchised depending on their shifting belonging to categories (i.e. ‘model’, ‘animal’, ‘humanity’) that organise ways of relating. It is particularly salient when biologists evoke the meaning of death in their activities. The hierarchy of deaths that biologists express is very often grounded in different ways of understanding life, as biology, as well as various embodied processes of being alive. Like there are different kinds of animals (Birke *et al*., 2007), there are different kinds of lives, which are expressed through the hierarchy of their deaths.

## Methods

I did a comparative ethnography in two developmental biology laboratories located in France, in the suburb of Paris and in Bangalore in the South of India. Between 2011 and 2014, I recorded interviews, and participated in the daily life of laboratories (Latour and Woolgar, 1979). I was invited to observe in cell culture facilities, at molecular benches and during lab meetings. During these occasions, I took notes of the conversations between the lab workers and discussed what they were doing with them. I also was given access to the written protocols in their laboratory notebooks. I also attended lunches and after work activities. In the following ethnography, four people were part of the laboratory located in France, and seven people were from a laboratory located in India. The quotations I reference in this article are from the two heads of laboratory, three Ph.D. candidates, one postdoc, one engineer, one student in internship, and two laboratory assistants. The French laboratory had a smaller team and no animal sacrifices were performed during my fieldwork. The biologists working in France referred to past experiences, before they entered their current laboratory where cell culture is the technique used to produce the molecular data of ‘life itself’ (Franklin, 2000).

I chose to do a comparative ethnography between a European based laboratory and an Asian based laboratory to study the reasons why human embryonic stem cells do not spark the same debates in those locations (Bharadwaj, 2012) and how scientific activities are performed by laboratory workers in the different contexts. This comparison shows that, despite these different contexts, common traits are to be found in such different locations. Biologists acknowledge local variations of debates about the manipulation of human embryonic stem cells, but they also express shared views. These shared views sustain the idea that scientific culture displays ‘global’ understandings of life (Franklin *et al*., 2000). This global culture of science refers to geographical, linguistic and ontological elements.

Similar scientific technologies can be found in both countries as well as similar languages (biological meanings expressed in English), either through the reading and writings of scientific articles, or through verbal communications. In France, I mainly communicated in French with biologists, though their meetings were conducted in English, as requested by the head of the laboratory, who wishes to train his team to the international language of their science (Houdart, 2008). In India, I mainly communicated in English with biologists, who commonly speak two to three languages (English and Hindi, the two national languages and very often several regional languages).

## Basic Science of Life Itself for Human Health

### Basic Science and Translational Outcomes

Death is an important topic when biologists from both laboratories talk about the translational (applicative) value of their basic science (Rapp, 2003; Friese, 2013). Even if the immediate outcomes of the research and articles published by the laboratories are not therapeutic, basic and translational research are linked together and united in a common goal.

Patrick, the head of the laboratory located in the suburb of Paris, has weekly ‘data sessions’ with his team to discuss the results of their experimental protocols. Even though his team is in France and they all speak French, Patrick requests that these sessions be done in English so that the researchers practice the shared language of the global scientific community. On one occasion, a biologist presented dissatisfying data. Patrick commented, in English: ‘We need something robust, we need to be sure of what we do. The patient is waiting for that.’ Indeed, the value and justification of basic research is to run experimentations on cells and animals allowing failures, toxicity and repetition of the same experimentation (Rémy, 2009; Thompson, 2013, chapter 6). Using cells makes sure of the safety of the future human patients who could be involved in clinical trials.^1^ In the same vein, Tamal, who works in the laboratory located in Bangalore as a Ph.D. candidate says:

There are two aspects again, one thing is the basic biology and the other one is the applicative biology, translational research. What I feel is that it needs to go hand in hand, because one cannot do without the other. Translational research is important, because it is ultimately going to lead to something, going to materialise into something, say a drug, or a vaccine, or a cure for a disease. That is definitely important. But to understand that, basic biology is again very important, so, without that you cannot understand the other.

If translational science cannot go without robust basic science, the latter is nonetheless strongly linked to applicative outcomes. Talking about the justification of their basic experiments, biologists in France and India refer to general categories such as ‘human’, ‘humanity’ and ‘society’ or ‘the public’ related to hope for new cures to be invented (McKay, 2000). These scientific projects are justified because patients belonging to the human kind (Birke *et al*., 2007) suffer and die from diseases.

For Padmini, the head of the laboratory in Bangalore, the value of huES is strongly connected to their translational potential: ‘What is valuable is what can be useful for the community, for curing’, she told me about cell lines. In basic research laboratories, the scope of knowledge is connected to ideas of being ‘useful to the general society’ so says Pratosh, a young intern student in India, as well as Sandeep, who is about to finish his Ph.D in the same institution. In terms of institution, these goals are also strongly underlined by the law or by the fund providers. In France, research using human embryonic stem cells must have a ‘therapeutic goal’ to be authorised.^2^ In India, this is not the case, but the medical horizon is nonetheless expressed as quite normative, especially if one wishes to get funding. Padmini, shared that: ‘When you open a lab, it is because you have a question to ask for the scientific community and for humanity, this is why you get funds.’

### Translational Outcomes through Life Itself

Basic science studies build on molecular understandings of life as being itself on global grounds (Franklin, 2000), as diseases are studied through their biology and associated to very general categories such as ‘humanity’. During an interview, Tamal talks about the purpose of doing research on the genetic mechanisms of cell development. He links the study of ‘life itself’ to its applicative potentials for ‘people’ who suffer from diseases associated with ‘genes’.

So eventually all the researches, according to me, should be directed in that how this is helpful to the human, mankind, genes, how it is helpful, how if you just knock down that gene, in case of cancer if you just obliterate the function of a gene, how the cancer is spread? How the cancer has progressed? Whether you can control the cancer … at least, if it cannot be controlled, whether you can understand the mechanisms of … so, then later on, people can get … take it off … 

Ouarda, a postdoctoral researcher in Patrick’s laboratory, had studied the genetics of a cardiovascular disease by comparing samplings of healthy and affected persons, when she was doing her PhD. In her interview she said: ‘I started by understanding why there is a cardiovascular disease, why you are affected by these diseases, and all, from the genetics’, and ‘for the postdoc, I have reached another level, which is to try to find solutions.’ Ouarda seemed to like her work a lot and was always very lively. When I asked her why her research is of so much interest to her, she answered: ‘I feel that I really do stuff for humanity’. ‘Can you tell me more?’ I added.

Because, she said, I try to participate, even if it is a small participation, it is really not that big compared to … when you find solutions and all. It is only a tiny step. But I know it is a key step to be able to do stuff for … 

Saying that, she vividly claps her hands, then apologised for that intense expression of her enthusiasm. ‘Sorry, to get to … for humanity, that’s it!’. Sandeep also evoked the therapeutic goal of research, circulated in one of the most renowned scientific journal that has an international reach, and narrates the happy end of a cure:

Yeah, I remember an article which is published in *Nature*. It was on the hematopoietic differentiation of stem cells and how these cells were transplanted into a bone marrow patient and the patient got recovered. Yes. It was a complete process and it had an ending. That was like a story for me, and it got me interested into it.

Biologists study basic biology of stem cells on a day-to-day basis and they continuously project a translational future, in which human patients are in need of treatments. In these projects, cells are materials for research. Biologists are aware that human embryonic stem cells can be associated with the destruction of human embryos (Testa, 2008). But, for the laboratory practitioners I talked to, the use of cells, and their destruction, are not associated with a form of death that needs particular recognition. When they discuss the destruction of embryos and the handling of human embryonic stem cells, death is not associated with genetics or biology more broadly, but with reflections on the differences of different kinds of lives, some deserving to have their deaths recognised and some not.

## HuES: Immortal Without a Body and a Soul

### HuES: Immortal or Dead, But Not Killed

Human embryonic stem cells are not considered to be particularly controversial for the heads of the laboratories I have been working with. Of course, they are nonetheless aware of the controversies and acknowledge them from the point of view of the location of their laboratories, while implying their personal and scientific views as well as the existence of a global scientific community who do not necessarily share the same views than other social groups on the status of the huES.

Padmini runs her laboratory in a country where huES research and human embryos *in vitro* manipulation do not spark debates about their status as potential individual human lives (Kumar, 2001; Bharadwaj and Glasner, 2009). It is easier for her to differentiate herself quite clearly from the western debates, associated with the USA, a country she considers ‘hypocritical’. In the process of distancing herself from American debates, she refers again to the death of ‘people’, whose lives are considered as more precious than the ones of the cells.

It is by discovering western opposition that I came to think about it. These stem cells … Prolife … For Indians, it seems really weird. Especially coming from a country a lot more violent and oppressive. The USA are bombing people. And then they have issues for these cells. It is hypocritical.

In comparison, Patrick runs his laboratory in a context where huES research is very controversial and has sparked a lot of public debates, like in the USA or many other countries in Europe. In France, huES research was banned until 2005 and banned with exceptional authorisations until it was authorised under exceptional circumstances in 2013. One of the reason is that human embryos are considered as ‘potential human persons’ by French bioethics, a status that define them as entities in between ‘bunches of cells’ and ‘persons’ (Merleau-Ponty, 2018b). Patrick explained why the term ‘line’ was so important to spread at the beginning of huES research – in the early 2000s. This term helped clarify the biologists’ views on these cells that are not understood as potential individuals who are destroyed but as a set of immortalised cells. In the following piece of interview ‘we’ refers to the community of researchers on a global scale, working within a western context in which it was important, in the 2000s, to differentiate embryos from cell lines in order to ease the controversies by stating a clear difference between the two. Patrick explained:

So this term of « line », was, indeed, [used] so that people understand well that, once we derived, once we isolated these human embryonic stem cells, these cells became immortal, because we had [cell] culture conditions allowing them to become immortal. This is what we call a line. And so, as soon as we had a line, we obviously did not need another embryo to obtain the same cells. And so, it seemed important because, very often, at least in the years 2000, people thought that, every day, when we wanted embryonic stem cells, we had, in quotation marks, “to kill” an embryo to obtain cells.

Using quotations marks is significant because it acknowledges ‘people’s views on embryos as potential individual lives while suggesting the idea that Patrick does not see the act of cell line derivation as a deadly one. In contrast, it is an act of immortalisation (which implications are not simple, see Lock, 2001 and Glasner, 2005). The idea that huES are not derived from an act of killing was also expressed when comparing the death of cells and the death of animals.

One day, Ouarda lent me her laboratory notebook from 2007. At that time she was a young unexperienced student doing an internship. In the notebook, I found a section dedicated to ‘the sacrifice of embryonic stem cells’, in which she describes diverse molecular protocols. I had never read or heard anybody refer to molecular protocols on cells as ‘sacrifices’, even if I understood what she was referring to. In order to study molecules implied in cell differentiation or pathological mutation, cells are cultured in petri dishes with diverse media containing molecules that interact with them while they develop over days or weeks in incubators with the right temperature and air composition (Landecker, 2007). Once the cell culture protocols are completed, the human and mouse cells are brought to the molecular benches where biologists mix the cells with diverse chemicals to extract DNA, RNA and proteins^3^ and study them. This intervention accompanies the disappearance of the cells. Once at the molecular bench, ‘it is dead’, comments Patrick.

Even if cells are dead at the molecular bench, writing that they are subjected to ‘sacrifices’ is not common at all. As I was quite surprised by this formulation, I asked Ouarda to explain it to me. She smiled and replied: ‘At this time, I thought like with animals, under the chemical hood, we kill them, we sacrifice them. I didn’t know.’ ‘And now, what do you say?’ I add. ‘Now’, she answers with a laugh, ‘I say that I do a cross-link!’. A crosslink is the process of chemically bounding two molecules present in the cells. In molecular biology, this technique is used to study structures and interactions of proteins.

Unlike ‘with animals’, lab animals, cells are not said to be killed, even if they can be ‘dead’ (Birke *et al*., 2007). Cells are processed by techniques. Indeed, when they are not used in molecular biology, biologists ‘throw’ the cells in a yellow biohazard bin dedicated to biomaterial waste. When cells are used for a molecular protocol, the name of the protocol is put forward. The act of transforming living cells in molecules is not associated with the idea of death, even if the cells are destroyed in the process. Of course, I am not referring to the biological knowledge of cell death here, also known as apoptosis, a mechanism understood as being co-constitutive to the life of cells (Landecker, 2003). I am pointing to the symbolical recognition accompanying the act of transforming living materials into data, a recognition that is associated with the use of terms like killing or sacrifices (Arluke, 1988; Lynch, 1988; Haraway, 1997; Rémy, 2009; Thompson, 2013).

Nonetheless, the absence of recognition to the fact that cells die when they are brought to the molecular bench does not mean that biologists do not care for their cells. Care and affect practices around cell cultures (Suzuki, 2015; Merleau-Ponty, 2018b) are not contradictory with their use as tools (Franklin, 2013; Meskus, 2018). And biologists can be devastated when their cells are contaminated, though, a hierarchy of deaths ease the tensions produced by the loss of cells. Blanche, a PhD student in Patrick’s lab mentioned a conversation she had with a friend of hers, a nurse working in a hospital who had put the life of a patient in danger because of a wrong dosage. Blanche explained: ‘I recall, I had called her because my cells were contaminated. She calmed me. She told me ‘No one died’ (*Il n’y a pas mort d’Homme*). It’s true.’

### HuES: A Kind of Life with No Soul

The acceptability to work on huES, and destroy them in the process, is sometimes related to the idea that they have no soul. Biologists who are believers can refer to non-secular (Roberts, 2016) understandings of life that they intertwine with biological approaches. Ouarda, does not feel that there is an issue with working with huES as she considers them as a ‘body without soul’, in accordance with her religion, Islam. Indeed, suras XXII, 5 et XXIII, 14–15 of the Qu’ran indicate how the embryogenesis unfolds in this context. The angel fixates the destiny of the child to be born after forty days of development. The individual soul is said to be present 120 days after conception (Walentowitz, 2003, p. 108). The fate of human embryos given to research is legitimate and Ouarda valorises their cells as materials for a therapeutic goal: ‘To me, a body without soul is nothing … well, nothing, these are cells we can use to cure other souls.’

Padmini, Patrick and Ouarda are not the only ones to dissociate individual lives and human embryonic stem cells. Blanche reaffirms the distinction between being alive and living a life, when she compares huES and induced pluripotent stem cells. Induced pluripotent stem cells are adult skin cells that are reprogrammed to resemble embryonic stem cells. They lose their skin identity and gain the capacity to differentiate into any body cell type. Blanche recalled the start of her research, when she thought about the status of her cells:

Clearly, it [the embryo] was such a premature stage that to me, the embryo, it was a bunch of cells and … careful, of course, things should not be out of control, I completely agree and all. But it was not an issue at all for me. (…) And to reprogram cells of someone who has lived … I am not a believer actually. There is something. Something happened. These cells, they lived. It was a living being. It was someone who thought, laughed, did a lot of things in their life. (…) I mean, there are populations who cry more for the death of elders that the death of newborns for example, because they lived, because they did a lot, even if it is the natural order of things … Well I would be a little … not like that, but I just say that from a biological perspective, if I want to draw a parallel, for me, the embryonic stem cell, the huES does not have a “soul” in quotation mark, when the iPS, it has a print somewhere.

This interview extract highlights a difference between living materials that are considered to have had a life because they belonged to actual persons, and ones that are not. This biological ‘print’ encapsulates an existence of thinking, laughing, and acting in a lifetime. This biological version of a ‘soul’, for this scientist who is not a believer, points at the lived life of someone who was alive and embodied. And this lived life gives more value to cell death than the one of cells that are alive, without having lived, like embryonic cells.

The emphasis on lived lives to make sense of death in the laboratories appears particularly salient when biologists talk about killing mice. Sacrifices, which entail the symbolical recognition of transforming a body into scientific data, appear to be connected to the recognition of living a life. This recognition differentiates mice’s lives from living cells. As mice are said to have a life of their own, their death is acknowledged and killing them is sometimes considered to be very difficult. The emotional hurdles that can arise during sacrifices are nonetheless eased through the overarching scientific and therapeutic goal, which make of mice lives living tools at the service of science and humanity and their biology.

## Reflections on Killing Mice

### Certain Ways to Take Lived Lives

Differentiating ‘cell culture’ from lab animals (Birke *et al*., 2007), Manon, a researcher in the French laboratory, stresses this shift from being alive (like cells) to having a life of one’s own, a life that is lived and experienced with other mice. Doing so, she used the term ‘killing’, by distinguishing it from being ‘contaminated’ and ‘thrown’ away, and she stressed the emotions that come with this important shift.

Manon: I have sacrificed, maybe, 350 mice, in three years of Ph.D., so after some time … I did it because I needed it for my studies, but it is not something I did with joy … I consider that I have worked enough on the animal for … I know that, I mean, I like other stuff, cellular culture for example. (…)

Noémie: Why do you prefer cell culture then?

Manon: Precisely because, for me, *you have less life in your hands*, so … .your cells are contaminated … well, *you throw them away, but it is not a life* [my emphasis]. But when you go get your cage … your mice, they are all well, rolled into a ball next to each other, sleeping. It is always a little annoying to kill them, so, euh … 

Noémie: It is more difficult.

Manon: Yes, it is. And me, what I liked too … at the beginning of my Ph.D., the first mouse I killed, really, it was a traumatism. I told myself that I could never do it … the problem is that, as we go along, you kill so many of them that, after some time, it is also mechanical, I mean that you do not realize … it is not that you do not realise, because me, I … when I knew I had a day of sacrifices, I went reluctantly. But you are less careful for the mouse you are killing than for the first one. It was more like an assembly line, and that, that bothered me after some time.

The emotional difficulty expressed is twofold. Firstly, the emotional difficulty relates to the idea that mice, on the contrary to cells, have their own life, shared with other mice. Secondly, sacrifices are difficult not only because of how mice’s lives are viewed, but also because their amount produces a situation in which the care implied in killing lessens with time, making them similar to products in an industrial setting. The association with the assembly line contradicts ideas of ‘killing well’ that are so important in ‘mortal love’ (Holmberg, 2011, p. 148). If killing well and avoiding objectification of these lives are important, mechanisms are nonetheless in place to restrain attachment (Daston, 1995). Karen is a research assistant in the Indian laboratory. The following quotation highlights Karen’s views on killing mice, and how different contexts of encounters are very important in how killings are experienced.

I cannot stand sacrificing a mouse, so I, most of the time, don’t do it myself. I ask for assistance. Especially the females, because I am of the same gender! (*She smiles*) I feel a little awkward to kill a female mouse but hey, I mean, in India, we have a lot of mice going here and there. (*laughs*) We … if I find a mouse in my home, I would definitely go with a broom to kill it. But these, because they are bred, and we get to them every day, they are more like our pets. So, but we haven’t … I haven’t named them, just numbered them (*laugh*).

Laboratory mice are closer to Karen than wild mice because she takes care of them, and, in the process, finds analogies between them and her. This caring relationship is similar to a domestic one, except that naming is not part of the picture – this is preventative measure taken to avoid becoming too attached to the animals. In other settings, analogies between humans and animals can be used to introduce moral sentiments in care practices. Lesley Sharp has written about biologists who work with primates. She argues that television is a means of humanising monkeys and a way to take care of them in a moral way. ‘If the use of macaques in science is to be a moral project, it requires the humanization of the monkey’ (Sharp, 2017, p.239). In Karen’s case, mechanisms are in place to avoid acknowledging similarities between herself and female mice. The absence of personification is a mechanism that allows for a certain emotional distance, but this distance is not complete and gender similarities produce an identification that makes it difficult to kill, and requires Karen to ask a colleague to do it for her.

The contexts through which animals are encountered are very important to navigate feelings and representations towards giving death and taking lives. The interpretation of laboratory contexts can be an occasion for societal debates. When I asked Manon if she had conversations outside of her laboratory about the daily handling of living entities in her activities, and especially human embryonic stem cells, she answered by pointing at anti animal experimentation activists that she had met in Nantes, a city of the West of France. She narrated how she could not agree with their depiction of death giving. She considers this approach to be hypocritical because it displays a slaughtering relationship between scientists and lab animals when, she argued, this type of killing is not the bloody type.

Manon: I remember an argument I had with … it was in Nantes, on the big square of Nantes. (…) There was an association campaigning against animal experimentation with things that really shocked me. An activist was seated, tied up to a chair with red all over the place to signify blood, with images … They showed atrocious images of animals’ mutilation and all. And me, I was in Ph.D., practicing on mice, so I had killed a lot in my activity and I found it, I mean, very hypocritical and really scandalous to show this when they do not know anything about it. So I went and told them … but, anyway, one cannot discuss with these persons. But this, I found it shocking (…) especially because, well, you show a mouse to the public, a rat being slaughtered, of course nobody likes to see that, so afterwards everybody is against animal experimentation. Yet, we could not have had some drugs without animal experimentation, so that’s it. As for stem cells, I had less discussions, I mean, questions about stem cells.

This extract summarises very well the hierarchy of deaths at play for some biologists, killing mice is not easy but is justified to develop new therapies, and the use of stem cells is not so much of a debate. Furthermore, animal death and death giving are indeed subjected to debates, fluidities, normativity and constructions that animal activists, ecofeminism or speciesism engage with by putting them on big squares. Manon criticises two things in anti-experimentation activism here, the wrong analogy and the wrong context. She criticises the instrumentation of other kinds of animals to consider the scientific sacrifices she thinks are legitimate, as hard as they are to perform, in the name of medicine and cure for a dying humanity.^4^

### ‘This Mouse is a Disease Model’

To ease the affective tensions implied in killing mice seen as lived lives, scientific training invites laboratory practitioners to see them as biological models, as tools used mechanically (Haraway, 2008). ‘We could not have had some drugs without animal experimentation’, finishes Manon to criticise anti-experimentation activism, even if sacrifices are not easy to perform. This dilemma at the heart of the ‘instrumentality-care continuum’ (Meskus, 2018) is also expressed by a laboratory assistant in the laboratory located in the suburb of Bangalore. Sadar referred to this dilemma by pointing at the recognition of death in the case of mice, because he thinks they have individual lives, endowed with a soul. But, a translational horizon for which they are disease models participates to the expression of a hierarchy of deaths.

No. Initially, when I had to sacrifice a mouse it was like … I was like ‘no I can’t kill it’. Like, adult mice, I can’t kill it. I can’t take anyone’s life, because I don’t like it, and also, I have no right to take anyone’s life. But like Doctor [Padmini, the head of the lab] said to me: […] “we are doing it for science. We are here to discuss something, something which will cure a disease, so for this, we need a disease model and *this mouse is a disease model* [my emphasis …] so you have to learn this.” So, initially, I didn’t like that much, like sacrificing a mouse because then you kill it, it will move for some time and then it will die. But slowly, slowly I got used to it.

Noémie: And now, it’s okay?

S.: Now, it’s okay.

N.: You don’t have any … 

S.: I’ll feel bad. Obviously, I’ll feel bad, but I’ll pray once for that mouse after killing it, but I have to kill.

N.: You’ll pray for the mouse?

S.: After killing, I will feel bad, I will say something, like … .

N.: Okay, okay. What will you say?

S.: I’ll just say that (*he smiles*) … Let its soul rest in peace. […] But I support this using mice as a model system, because you need something to study the diseases. And then, if you don’t study the disease, then obviously it will affect many people and many people will die.

To be a lab animal, in this case, is not only to be made a disease model that is cared for, for the making of proper science, but also to be recognised as having a life of one’s own that is to be respected and the capacity to be given a death, which affectively costs the one who kills. This emotional price will be less and less with time, as the animal is seen less as an embodied individual with a soul and is more directly considered as a disease model used to help human lives.

I also met several biologists who do not pay the same emotional price. The interview from which the following moment is extracted was done after several interviews during which I heard about the emotional difficulties to sacrifice mice. I was very cautious then, implying that it was difficult for my interlocutor too. I was wrong. Indeed, Balaji, also a PhD student in Padmini’s lab, did not initially understand my question because his focus was on the material he studies, not its source. In his case, the scientific training is dominant in his understanding of life valuation. In his research, Balaji uses embryos dissected from gestating mice at different stages of development. In order to access the embryos, he sacrifices the animals by rupturing their neck before they are dissected to extract the gestational sacs in which the materials of study are located.

Noémie: Let’s go back to the mice, is this something, euhhhh, that you … How can I say that? Euh, what is it for you to have to sacrifice mice for your research?

Balaji: Embryos … 

N: And mice, because you have to kill the mice for … 

B: For getting embryos … 

N: Is this something that never gave you any problem, or you had to think about it or … ? You know. You did this for the first time for your Ph.D or before?

B: Yes for the first time for my Ph.D but … truly speaking it didn’t give me much … much pain that we are killing a mouse.

N: Do you have any idea why?

B: If we kill a mouse and we don’t get the embryos then we think ‘oh … we … useless … sacrificed this mouse’. But, in general, if we get embryos out of it and we are able to do … and if we get something new, then it is for better … […] It is for more knowledge.

Even when killing does not bear an emotional price, this act is still called a deadly one, a sacrifice of an animal, and not ‘doing a cross link’, like with human embryonic stem cells brought at the molecular bench. The recognition of the lived lives is associated with the idea that sacrifices should be useful. The overarching rationale is that science and knowledge assist the pursuit of making potential cures.

## Conclusion

How do developmental biologists talk about death in their activities? What does death reveal about the ways biologists understand the lives of the materials they work with (cells and mice)? Different expressions of deaths are to be found relating mice, cells and patients in a hierarchy that points at different kinds of lives.

The sacrifice in experimental settings has been extensively studied, pointing at one expression of death implying the process through which lab animals are transformed into proper data for the sake of science and medical translation (Arluke, 1988; Lynch, 1988, Haraway, 1997; Birke *et al*., 2007; Rémy, 2009; Thompson, 2013). In both laboratories, professionals express how the suffering and death of human patients is an overarching compass, through biological traits of ‘life itself’ (Franklin, 2000, p. 190). The notion of ‘life as such’ has been created to discuss the biologisation of life and to point at lives that are lived and embodied, in the context of migration (Fassin, 2009, p.48). The ethnography of this text invites the suggestions offered by the notion of life as such into biology laboratories where life itself is studied and made (on IVF see also, Merleau-Ponty, forthcoming and Bärnreuther, 2018). It is through the acknowledgments of different forms of death that we have shown this, highlighting a hierarchy justified by various ways biologists understand what ‘life’ means.

Birke *et al*. (2007, p. 19) have shown how animals belong to different ‘kinds’ when relating to humans. ‘Sacrifices’ of ‘lab animals’ are transformative processes that entail the identification and differentiation of the animals from their human counterparts, both biologically and morally (Haraway, 2008, p. 79). The study of cells’ use show common traits, such as ‘the continuum of instrumentality and care’, the expression of ‘mortal love’ in handling these materials, or their transformation into molecular data (Holmberg, 2011, p. 148 and 154; Meskus, 2018, chapter 5). But, the ethnography presented here shows that these similarities between animals and cells’ uses do not lead to calling cell death an act of ‘killing’ or ‘sacrifice’.

Even if laboratory practitioners are well aware of the controversies associated with ‘destroying’ human embryos to experiment on their stem cells, whether they work in France or India, they are not personally attached to such characterisation. Cells are not said to be ‘sacrificed’, even if they are cared for and they lose life when their molecules are analysed after cell cultures, when molecular data are extracted from them. The death of cells is not recognised beyond its technical and biological definition. This absence of recognition points to the idea that these cells do not have a life that is lived, on the contrary to mice. Comparing cell death and mice death give another perspective on sacrifices, that are not only about transforming animals into data through a continuum of instrumentality and care, identification and differentiation, but also about acknowledging lives that are lived, even if they are taken for science, humanity and hopefully, human patients.

Compared to the absence of symbolical recognition of cell death, the death of mice is strongly acknowledged through expressions of death giving like ‘killing’ or ‘sacrifices’, more or less intense emotions, reluctance and actions that underline the importance to respect those lives through care in giving death, and sometimes, even prayers for their souls. Nonetheless, mice are lab animals, and scientific culture has ways of building the acceptance of their deaths, by associating them with tools, disease models, and, doing so, by reaffirming the overarching death of all, the ones of human patients. Mice, like humans, have a life of their own, but they are lab animals, which put their death at the service of translational outcomes.

Life as a set of molecules and their various interactions in the early days of embryonic development is what the two laboratories I have investigated study. The daily practice of science narrated by the laboratory workers in my ethnographical study enact other perceptions that point at being alive or having a life, at lives that are said to be embodied and experienced, while being hierarchised and made unequal in their treatments, for the sake of science and humanity. Inspired by the idea of ‘kinds of animals’, (Birke *et al*., 2007), I suggest that a *hierarchy of deaths* between cells, mice and patients is a fine expression of different *kinds of lives* in two laboratories of developmental biology.

## Notes

1 The use of animals in basic research is connected to the rise of bioethics after the Second World War and the Nuremberg trials. (Löwy and Gaudillière, 1998; Gaudillière, 2001).

2 (article L2151-5 of the public health code).

3 DNA (deoxyribonucleic acid): a biological macromolecule with a double helix shape. It is present in all cells and inside some viruses. It is the material support of genetic information, or genome, from which proteins necessary for cell functions are synthetised. To do so a piece of the DNA is translated into RNA. RNA (ribonucleic acid): Biological molecule, a copy of a piece of DNA (a gene). It helps connect together, following a specific order, some molecules that are present in the cell thanks to protein degradation or alimentation. It helps the synthesis of proteins. Protein: Biological macromolecule having a particular function in a cell. Molecule: Basic structure of matter that is constituted of the chemical assemblages of atoms.

4 Indeed, the work implied in slaughtering animals, in the case of food production is yet another type of killing animals (Vialles, 1994).
